# Big Data in der Gesundheitsförderung und Prävention: Ergebnisse einer Delphi-Studie mit einem Schwerpunkt auf vulnerable Gruppen

**DOI:** 10.1007/s11553-021-00871-8

**Published:** 2021-07-01

**Authors:** Julia Spranger, Marlen Niederberger

**Affiliations:** Forschungsmethoden in der Gesundheitsförderung und Prävention, Pädagogische Hochschule Schwäbisch Gmünd, Oberbettringer Straße 200, 73525 Schwäbisch Gmünd, Deutschland

**Keywords:** Digitale Datenmengen, Gesundheit, Vulnerable Gruppen, Delphi-Verfahren, Expertenbefragung, Digital data volume, Health, Vulnerable groups, Delphi technique, Expert survey

## Abstract

**Hintergrund:**

Die Nutzung großer und vielfältiger Datenmengen (Big Data) kann zur Gewinnung gesundheitsbezogener Erkenntnisse führen. Die Relevanz untermauern aktuelle Erfordernisse, bspw. in Zusammenhang mit der Digitalisierung, der Gesundheitsversorgung in Ausnahmesituationen und der zunehmenden Bedeutung von Personalisierungsprozessen in der Gesundheitsforschung. Das Potenzial von Big Data zur Erforschung vulnerabler Gruppen ist strittig, jedoch vor dem Hintergrund relativ stabiler sozialbedingter gesundheitlicher Ungleichheit besonders relevant.

**Ziel der Arbeit:**

In der Studie wird untersucht, wie Expert*innen im Bereich der Analyse von Gesundheitsdaten das Potenzial von Big Data in der Gesundheitsförderung und Prävention, insbesondere zur Erforschung vulnerabler Gruppen, einschätzen.

**Material und Methode:**

In einer Delphi-Studie wurden Expert*innen in zwei Runden mit einem Onlinefragebogen befragt, um Konsens und Dissens über das Potenzial von Big Data zu identifizieren.

**Ergebnisse und Schlussfolgerung:**

Aus Sicht der Expert*innen birgt Big Data ein Potenzial für die Gesundheitsförderung und Prävention, insbesondere im klinischen Setting und durch die Personalisierung gesundheitsbezogener Maßnahmen. Vor allem Menschen mit seltenen Erkrankungen und ältere Personen könnten durch Big-Data-Analysen profitieren, bspw. durch beschleunigte Diagnoseprozesse oder personalisierte digitale Gesundheitsanwendungen. Uneinig sind sich die Expert*innen über den Umfang, in welchem es Forschungseinrichtungen, Krankenversicherungen oder Unternehmen, erlaubt sein soll, derartige Daten zu nutzen oder zu teilen.

## Abstract (en)

**Background:**

The use of large and diverse amounts of data (Big Data) can lead to the gaining of health-related insights. The relevance is underlined by current challenges, for example in connection with digitization, health care in exceptional situations and the increasing importance of personalization processes in health research. The potential of Big Data for research on vulnerable groups is controversial, but particularly relevant against the background of relatively persistent socially determined health inequalities.

**Objectives:**

The study examines how experts in the field of health data analysis assess the potential of Big Data in health promotion and prevention, especially for research on vulnerable groups.

**Materials and methods:**

In a Delphi study, experts were surveyed in two rounds using an online questionnaire to identify consensus and dissent on the potential of Big Data.

**Results and conclusions:**

From the experts’ point of view, Big Data holds potential for health promotion and prevention, especially in the clinical setting and in the personalization of health-related measures. People with rare diseases and older people could benefit from Big Data analyses, for example through faster diagnostic processes or personalized digital health applications. The experts disagreed on the extent to which research institutions, health insurers or companies should be allowed to use or share such data.

Big Data ist interdisziplinar ein hochaktuelles Thema. In der Gesundheitsförderung und Prävention besteht noch Unklarheit über das Potenzial von Big Data zur Generierung neuer Erkenntnisse, insbesondere in Bezug auf die Erforschung vulnerabler Gruppen. In diesem Beitrag werden die Ergebnisse einer Delphi-Studie zu diesem Thema vorgestellt. Befragt wurden Expert*innen im Bereich Big Data und Gesundheit aus Wissenschaft, Wirtschaft und Politik.

## Hintergrund und Fragestellung

Unter dem Schlagwort Big Data werden im Gesundheitsbereich große digitale Datenmengen sowie verschiedene Datenquellen und -strukturen diskutiert [2, 6, 15, 27]. Zwei Bedingungen, die maßgeblich zur Profilierung von Big Data beigetragen haben, sind der gesunkene Preis für Datenspeicherplatz sowie leistungsfähigere Computersysteme [17]. Sich stets ändernde Möglichkeiten der digitalen Datenerhebung und -analyse sowie unpräzise Formulierungen (bspw. was „große“ Datenmengen sind) erschweren eine klare oder allgemeingültige Definition von Big Data [17, 34]. Oftmals wird Big Data über die drei V, den Datenumfang („volume“), die Datenvielfalt („variety/variability“) und die Geschwindigkeit der Datengenerierung/-transferierung bzw. -auswertung („velocity“) charakterisiert [2, 21]. Der Erkenntnisgewinn beruht nicht nur auf der Datenmenge („volume“), sondern ergibt sich v. a. aus der Kombination von etablierten, analogen und neuen digitalen Datenquellen („variety/variability“; [2, 15]). Die Geschwindigkeit („velocity“) der Analyse und Auswertung wird in Echtzeit angestrebt [2]. Die drei V „volume“, „velocity“ und „variety“ sind auf einen Bericht aus dem Jahr 2001 von Doug Laney, ein Analyst eines weltweiten Marktforschungs- und Beratungsunternehmens (Gartner), zurückzuführen [18]. Im Gesundheitsbereich werden mittlerweile insbesondere die Datenqualität bzw. Verlässlichkeit („veracity“) als viertes V diskutiert. Diese bestimmt, wie valide und reliabel die Aussagen über medizinische Prognosen oder Erkenntnisse zu Therapie- und Präventionsmaßnahmen sind [2, 27]. Der Hype um das Schlagwort Big Data begann nach Daten von Google-Trends um 2010, v. a. zur Erfassung und Analyse von Unternehmenskennzahlen [17].

Der Einsatz von Big Data im Gesundheitsbereich wird maßgeblich von drei verschiedenen Trends bestimmt:

- Das Thema Gesundheit nimmt in der Gesellschaft einen zentralen Stellenwert ein [23]. Moderne Technologien, wie Wearables oder Gesundheits-Apps, tragen dazu bei, dass Personen gesundheitsbezogene Faktoren, wie bspw. Schrittzahl, Herzschlag oder UV-Strahlung, selbst überwachen und die Ergebnisse zur Verbesserung des Gesundheitsverhaltens und auch langfristig zur Verbesserung des Gesundheitszustands nutzen können [1].
- Der demographische Wandel prägt und verändert die Anforderungen an das Gesundheitswesen insbesondere in der Gesundheitsversorgung und Pflege [2, 14]. Neue Technologien und Datenzugänge unterstützen die Digitalisierung im Gesundheitswesen [2]. Eng damit zusammen stehen Versprechungen an eine verbesserte Versorgungsqualität und eine gesteigerte Versorgungseffizienz, insbesondere für Ältere [6, 7].
- Anforderungen von Patienten*innen an personenbezogene Therapie- und Präventionsmaßnahmen nehmen zu, weshalb die Notwendigkeit der Nutzung umfassender personenbezogener Daten durch Behandelnde und Pflegende zunehmend relevant wird [2, 32].

Big Data wird in der Gesundheitsforschung momentan insbesondere unter technischen, datenschutzrechtlichen und anwendungsorientierten Aspekten diskutiert bzw. erforscht. Wichtige Schlagworte sind: Datengrundlagen und -verarbeitung [3, 6, 21, 27], Methoden zur Analyse und Visualisierung [6, 20], Data Literacy [16, 25], Effizienzsteigerung [1, 2, 35], Ethik und Zugang [10, 24, 27, 35], Risikoanalyse und Prognose [3, 12, 28, 30, 33].

Verschiedene Studien belegen das Potenzial von Big Data im Gesundheitsbereich. So konnte über die Datenanalyse in einer pharmakoepidemiologischen Forschungsdatenbank (GeParD) das Risiko eines Fieberkrampfes durch einen Impfstoff bei Kindern unter 5 Jahren ermittelt und so die Qualität in der Impfversorgung von Kleinkindern in Deutschland verbessert werden [28]. Weitere positive Erfahrungen belegen Studien zu psychischen Erkrankungen, wie unipolare Depression, Schizophrenie oder Autismus-Spektrum-Störungen [30]. Durch Analysen in gesundheitsbezogenen Datenbanken gelang es, neue Informationen über Risikofaktoren, Medikation und Prognose zu gewinnen, wobei die nationale Datenlage stark variiert und internationale Vergleiche erschwert [30]. Auch im Zuge der COVID-19-Pandemie („coronavirus disease 2019“) werden Big-Data-Analysen genutzt, um durch Tracking von positiven Fällen die Ausbreitung des Virus zu modellieren und Prognosen zu erstellen [33].

Ob und ggf. wie Big Data zur Erforschung vulnerabler Gruppen beitragen kann, wird kontrovers diskutiert. Kritisch gesehen wird z. B. der derzeit vorherrschende Fokus auf biomedizinische Daten als Input für Big-Data-Analysen oder die Gefahr der Ausgrenzung durch das Nicht-Vorhandensein oder -Einbeziehen digitaler Daten von bestimmten Gruppen (Populations‑/Sampling-Bias bzw. algorithmischer Bias) [7, 8, 24]. Unter Gesichtspunkten relativ stabiler sozialbedingter gesundheitlicher Ungleichheit sind spezifische Gruppen, wie bspw. Menschen mit Migrationshintergrund, Alleinerziehende oder Arbeitslose, für die Gesundheitsförderung und Prävention jedoch hochrelevant [31]. Vulnerabilität zeigt sich hier durch vergleichsweise hohe Erkrankungs‑, Behinderungs- und Sterbewahrscheinlichkeiten und einem erschwerten Zugang zur Gesundheitsversorgung [29]. Zudem gelten vulnerable Gruppen als schwer erreichbar und „selten gehört“ sowohl für die Forschung als auch für die Praxis von Gesundheitsförderung und Prävention [26, 36]. Das Potenzial von Big Data in der Forschung der Gesundheitsförderung und Prävention, insbesondere für vulnerable Gruppen, wurde in einer Delphi-Studie aus dem Blickwinkel einschlägiger Expert*innen untersucht. Big Data wurde dabei als die Nutzung, großer und vielfältiger digitaler Datenmengen zur Gewinnung neuer Erkenntnisse definiert [17, 35].

## Methode

Delphi-Verfahren sind mehrstufige, strukturierte Befragungsverfahren. Ziel ist es, die Einschätzungen und das Wissen von Expert*innen systematisch über mehrere Runden zu erfassen, meist mit dem Ziel Konsens in den Urteilen zu erhalten. Das Besondere ist, dass die Expert*innen ab der zweiten Runde ein Feedback über die Ergebnisse der vorherigen Runde erhalten und so ihre Urteile überdenken können [9]. Die Delphi-Methode ist ein etabliertes Verfahren zur Einschätzung wissensbasierter Urteile von Expert*innen mit dem Ziel der Identifikation von Konsens bzw. Dissens [9, 22].

**Abb. 1:**
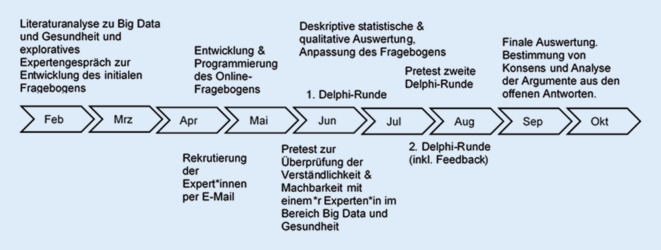
Überblick über das methodische Vorgehen im Zeitverlauf des Jahrs 2020 (eigene Darstellung)

In der vorliegenden Delphi-Studie wurden die Expert*innen in zwei Delphi-Runden mit einem Onlinefragebogen befragt. Ein Überblick über das methodische Vorgehen gibt Abb. 1. Ziel war es, Konsens in den Urteilen zu erhalten. Die Konsenskriterien wurden vorab festgelegt (Tab. 1). Die Festlegung von 70 % Zustimmung als Konsenskriterium ist dabei typisch für Delphi-Verfahren in den Gesundheitswissenschaften [22].

**Tab. 1:** Kriterien zur Definition von Konsens (eigene Darstellung)

Skala	Konsenskriterium
5‑stufige Ratingskalen	Interquartilsabstand (IQR) ≤ 1
Dichotome, 4‑stufige Ratingskalen und Rankingskalen	70 % Zustimmung

### Erste Delphi-Runde.

Der initiale Fragebogen wurde auf Basis einer umfangreichen Literaturanalyse und eines explorativen Expertengesprächs entwickelt. Inhaltlich gliederte sich der Fragebogen in fünf Bereiche:

1. Big Data in der Gesundheitsförderung und Prävention,
2. Potenzial von Big Data zur Erforschung vulnerabler Gruppen,
3. Zukunftsszenarien,
4. Angaben zu Person und Expertise,
5. offene Anmerkungen.

Der Fragebogen enthielt 37 Fragen, wobei die Mehrheit der Fragen, abzüglich der 8 Fragen zur Expertise der Person, offen waren (*n* = 16/29 Fragen). Die offenen Fragen wurden in Anlehnung an die thematische Analyse von Braun und Clarke (2006) und die geschlossenen Fragen deskriptiv mit R ausgewertet [4]. Ziel war es, aus den offenen Fragen standardisierte Items für die zweite Delphi-Runde zu entwickeln und bei den geschlossenen Items Konsens zu identifizieren (vgl. Tab. 1).

### Zweite Delphi-Runde.

Die Items, bei denen kein Konsens erzielt wurde (*n* = 7), sowie die neu entwickelten Items (*n* = 72) wurden in der zweiten Runde zur Bewertung gestellt. Diese beinhaltete insgesamt 36 Fragen. Als statistisches Feedback wurden die eigene Antwort aus der vorherigen Runde sowie eine Häufigkeitsverteilung aller Antworten, mit Angabe des Mittelwerts und der Standardabweichung, zu jedem Item angegeben. Auf Basis dieser Angaben konnten die befragten Expert*innen ihr Urteil bestätigen oder anpassen.

Aufgrund von zeitlichen Ressourcen war die Anzahl der Delphi-Runden auf zwei Runden determiniert.

### Stichprobe

Als Expert*innen wurden Personen aus Wissenschaft, Wirtschaft, Politik und Gesundheitswesen ausgewählt, die über Fachwissen im Bereich Big Data und Gesundheit verfügen und in diesem Bereich in Deutschland einschlägig tätig sind bzw. waren. Die Identifikation erfolgte über Publikationen, Vorträge und entsprechende Forschungsprojekte. Zudem wurden über die freie Suche im Internet oder im Zuge der analysierten Literatur relevante Unternehmen und Startups sowie wissenschaftliche Einrichtungen aus dem Themenfeld über die geplante Delphi-Studie informiert und um die Nennung von Expert*innen gebeten.

Von den letztendlich 105 eingeladenen Expert*innen meldeten sich etwa die Hälfte zurück. 20 Personen haben an der ersten und von diesen wiederrum 16 Personen an der zweiten Delphi-Runde teilgenommen. Tab. 2 gibt einen Überblick über die Zusammensetzung der Stichprobe.

**Tab. 2:** Fragen zur Person und Expertise (eigene Darstellung)

		Runde 1 (*n* = 17^a^)	Runde 2 (*n* = 16)
*Fragen zur Expertise*			
Erfahrungsjahre	MW (SD)	8,2 (±6)	8,4 (±6,1)
Kenntnisse über technische Aspekte zu Big Data (*n* [≈%])	Sehr gut	3 (18 %)	3 (19 %)
	Gut	7 (41 %)	7 (44 %)
	Grundlegend	7 (41 %)	6 (38 %)
Kenntnisse über gesundheitliche Aspekte von Big Data (*n* [≈%])	Sehr gut	9 (53 %)	7 (44 %)
	Gut	8 (47 %)	9 (56 %)
Kompetenz im Bereich Gesundheitsförderung und Prävention (*n* [≈%])	Sehr hoch	6 (35 %)	5 (31 %)
	Hoch	4 (24 %)	3 (19 %)
	Durchschnittlich	6 (35 %)	7 (44 %)
	Niedrig	1 (6 %)	1 (6 %)
*Fragen zur Person*			
Berufsgruppe (*n* [≈%])	Wissenschaft	11 (65 %)	11 (69 %)
	Politik	1 (6 %)	1 (6 %)
	Gesundheitswesen	2 (12 %)	2 (13 %)
	Wirtschaft	3 (18 %)	2 (13 %)
Alter (Jahre)	MW (SD)	45,9 (±12,3)	46,3 (±12,6)
Geschlecht (*n* [≈%])	Männlich	13 (76 %)	12 (75 %)
	Weiblich	4 (24 %)	4 (25 %)

*MW* Mittelwert, *SD* Standardabweichung, *n* Anzahl der Personen, die diese Frage beantwortet haben

^a^Drei Personen haben die Fragen zur Person in der ersten Runde nicht beantwortet

## Ergebnisse

Insgesamt wurde für ca. 50 % aller standardisierten Items am Ende der zweiten Delphi-Runde, mit einer fünf- oder zweistufigen Skala, Konsens zwischen den Expert*innen erzielt.

### Big Data in der Gesundheitsförderung und Prävention

Die Expert*innen wurden nach dem Potenzial verschiedener gesundheitsbezogener Daten für Big-Data-Analysen zur Gesundheitsförderung und Prävention gefragt (Tab. 3). Einig sind sich die Expert*innen über das grundsätzliche und vielfältige Potenzial. Für medizinische Daten und Forschungsdaten allgemein sehen die Befragten gemessen am Mittel das höchste Potenzial. Am geringsten schätzen sie die Einsatzmöglichkeiten nichtklassischer Gesundheitsdaten für die Gesundheitsförderung und Prävention ein. Der Umfang, in welchem es Forschungseinrichtungen, Krankenversicherungen oder Unternehmen erlaubt sein soll, gesundheitsbezogene Daten für Analysen zu nutzen, blieb auch in der zweiten Delphi-Runde strittig. Als zukünftigen Einsatzbereich identifizieren die Expert*innen v. a. den klinischen Bereich, bspw. die bildgestützte Diagnostik durch künstliche Intelligenz (KI) und die Personalisierung gesundheitsbezogener Interventionen, bspw. auf Basis von Nutzerdaten generiert durch Wearables oder Smartphones.

**Tab. 3:** Einschätzung des Potenzials gesundheitsbezogener Daten. Ergebnisse der ersten und zweiten Delphi-Runde (eigene Darstellung)

Wie schätzen Sie das Potenzial der folgenden Datentypen für Big-Data-Analysen zur Gesundheitsförderung und Prävention auf einer Skala von 1 (sehr niedrig) bis 5 (sehr hoch) ein?			
Item	MW (SD)	IQR	Konsens
Medizinische Daten (z. B. Laborbefunde, Genomik, Patientenprofile)	4,8 (±0,5)	0	*Ja*
Versicherungsdaten (z. B. Versicherungsinformationen, Risikoprofile)	4,2 (±0,8)	1	*Ja*
Forschungsdaten (z. B. klinische Versuche, Open Data)	4,5 (±0,6)	1	*Ja*
Individuelle, durch Nutzer generierte Daten (z. B. Ernährung, Fitness, Sensoren)	3,9 (±1)	1	*Ja*
Pharmadaten (z. B. Verkauf und Zusammensetzung von Medikamenten, Beschwerden)	4 (±0,7)	0,5	*Ja*
Nicht-klassische Gesundheitsdaten (z. B. von sozialen Netzwerken, Verkehrsdaten)	3,5 (±1,2)	1	*Ja*
Öffentliche Gesundheitsdaten (z. B. von Gesundheitsämtern, Nachgelagerten Behörden)^a^	3,9 (±1,1)	2	Nein
Sozioökonomische Daten (z. B. Herkunft, Einkommen)^a^	4,1 (±0,8)	0,5	*Ja*

Skala: 1 sehr niedrig, 2 niedrig, 3 teils/teils, 4 hoch, 5 sehr hoch; Teilnehmerzahl: N_R1_ = 20 (Runde 1), N_R2_ = 16 (Runde 2)

*MW* Mittelwert, *SD* Standardabweichung, *IQR* Interquartilsabstand

^a^Ergebnisse der zweiten Delphi-Runde

Die Expert*innen wurden nach notwendigen Bedingungen für Big-Data-Analysen im Gesundheitsbereich gefragt. Alle Expert*innen stimmen zu oder eher zu, dass ein klarer Projekt- bzw. Forschungsauftrag vorausgesetzt und das Erhebungsverfahren der Daten auf ethische Unbedenklichkeit geprüft werden muss. Zudem sind sie sich einig (*n* = 15/16 [≈94 %]), dass Daten zur Analyse anonymisiert oder pseudonymisiert werden müssen. Gleichzeitig räumen sie ein, dass der Erhalt einer Zustimmung von Nutzern*innen zur Datenverarbeitung herausfordernd ist. Dieser Aussage stimmen *n* = 10/16 (≈63 %) der Expert*innen zu oder eher zu. Dahinter steht möglicherweise die Uneinigkeit der Expert*innen, ob die Kompetenz von Patient*innen bzw. Kund*innen ausreicht, um eine informierte Entscheidung zur Datenverarbeitung zu geben. Für wichtig halten die Expert*innen die Schulung und Förderung der digitalen Bildung und Gesundheitskompetenz in der Bevölkerung (*n* = 13/16 [≈81 %]).

### Big Data in der Gesundheitsförderung und Prävention vulnerabler Gruppen

Rund 79 % (*n* = 11/14) der Expert*innen sehen in Big Data einen Mehrwert für die Forschung in der Gesundheitsförderung und Prävention. 83 % (*n* = 15/18) sehen speziell für die Gesundheitsförderung vulnerabler Gruppen ein Potenzial. Offen genannte Gruppen sind:

- Menschen mit seltenen oder chronischen Erkrankungen,
- physisch oder psychisch beeinträchtigte (ältere) Personen,
- diskriminierte und marginalisierte Gruppen.

Zwischen den Expert*innen bleibt jedoch strittig, inwiefern sich das Potenzial von Big Data zur Gesundheitsförderung und Prävention zwischen vulnerablen und nicht-vulnerablen Gruppen unterscheidet und ob partizipative Forschungsmethoden in der Erforschung vulnerabler Gruppen einen geeigneteren Ansatz bilden oder Big-Data-Analysen mit anderen Ansätzen kombiniert werden müssten (Tab. 4).

**Tab. 4:** Big Data in der Gesundheitsförderung und Prävention vulnerabler Gruppen und nicht-vulnerabler Gruppen. Ergebnisse der zweiten Delphi-Runde (eigene Darstellung)

Im Folgenden wird auf das Potenzial der Nutzung von Big Data als Instrument zur Gesundheitsförderung und Prävention für vulnerable Gruppen eigegangen. Stimmen Sie den Aussagen zu?			
Item	MW (SD)	IQR	Konsens
Big Data bietet einen Mehrwert für die Gesundheitsförderung und Prävention	4,1 (±1)	1	*Ja*
Die Chancen, Risiken und Anwendungsbereiche von Big Data in der Gesundheitsförderung und Prävention unterscheiden sich zwischen vulnerablen und nicht-vulnerablen Gruppen	3,8 (±1,3)	1,5	Nein
Vulnerable Gruppen profitieren von partizipativen Forschungsansätzen mehr als von der Forschung durch Big Data	3,3 (±1,4)	1,5	Nein

Skala: 1 stimme nicht zu, 2 stimme eher nicht zu, 3 teils/teils, 4 stimme eher zu, 5 stimme zu

*MW* Mittelwert, *SD* Standardabweichung, *IQR* Interquartilsabstand

In der ersten Delphi-Runde hatten die Expert*innen die Möglichkeit, offen die Chancen und Risiken von Big Data für die Gesundheitsförderung und Prävention vulnerabler Gruppen zu formulieren (Tab. 5). Als Chance wird die frühzeitigere, effizientere und durch die umfassende Datenbasis fundiertere Identifikation von gesundheitlichen Risikofaktoren gesehen und die Möglichkeit, effektivere Interventionen zu entwickeln, die viele Personen, u. a. auch vulnerable Gruppen und herausfordernde Settings mit komplexen Strukturen und unterschiedlichen Akteuren, erreichen. Inwiefern die Chancen von Big Data im Gesundheitsbereich greifen können, hängt nach Einschätzung der Expert*innen maßgeblich von der Akzeptanz in der Bevölkerung ab. Einige der Expert*innen erwarten, dass durch die Corona-Warn-App grundlegende Weichen für die Big-Data-Akzeptanz in der Bevölkerung gestellt werden, auch in Bezug auf den Datenschutz.

**Tab. 5:** Offene Antworten der Expert*innen zu Chancen und Risiken von Big Data für vulnerable Gruppen (Ergebnisse der ersten Delphi-Runde, eigene Darstellung)

Chancen	Risiken
Prävention, z. B. durch frühzeitige Identifikation protektiver Faktoren und Verhaltensweisen	Intransparenz, z. B. durch mangelnde Nachvollziehbarkeit von Algorithmen
Effizienzsteigerung, z. B. durch schnellere und automatisierte Abläufe	Verlust persönlicher Kontakte, z. B. von Vertrauensbeziehung zwischen Patient*innen und Pflegenden
Rationalisierung von Forschungs- und gesundheitlichen Versorgungsprozessen, z. B. Diagnostik, sowie politischen Entscheidungen, auch in gesellschaftlichen Ausnahmesituationen, z. B. während der COVID-19-Pandemie	Datenmissbrauch und -kontrolle
Effektivere Entwicklung und verbesserte Evaluation von Interventionen	Falsche Schlüsse, z. B. durch Fehlinterpretation von Korrelationen als kausale Effekte
Erschließung weiterer Zielgruppen, z. B. bisher schwer zugängliche Gruppen	Verlust der informationellen Selbstbestimmung
Analyse komplexer Forschungsfragen, z. B. bei multiplen Kontextfaktoren, mehreren Versorgungsebenen, unsteten Beziehungsgefügen, fehlenden Zugängen für Befragungsstudien oder wenn Studien hoher Evidenzgrade nicht einsetzbar sind	Kein Ausschöpfen des Potenzials von Big Data, z. B. durch hohe Datenschutzhürden und Kompetenzansprüche an Forschende
–	Stigmatisierung und Ausgrenzung spezifischer Gruppen, z. B. bei Verzerrungen der Population (Populations‑/Sampling Bias) oder durch den Algorithmus (Algorithmic Bias)

*COVID-19* „coronavirus disease 2019“

Bei der Bewertung der Chancen und Risiken von Big Data für vulnerable Gruppen sind sich die Expert*innen einig, dass diese ausgeglichen sind (*n* = 4/17 [≈24 %]) oder die Chancen sogar überwiegen (*n* = 11/17 [≈65 %]).

## Diskussion

Die Expert*innen sehen in Big Data einen Mehrwert für die Gesundheitsförderung und Prävention, bspw. zur bildgestützten Diagnostik in Zusammenhang mit künstlicher Intelligenz, zur Entwicklung von personalisierten Interventionen oder zur Evaluation von digitalen Gesundheitsanwendungen. Die Expert*innen halten es für wahrscheinlich, dass Umgebungen durch Big-Data-Analysen auf eine gesundheitsförderliche Weise angepasst und spezifische Interventionen, insbesondere im Bewegungsbereich, ausgearbeitet werden können, die erfolgreicher in der Umsetzung sind als bisherige Angebote. Andere Studien warnen aber, dass Bürger*innen durch Big-Data-Analysen eine permanente Überwachung befürchten (Big-Brother-Narrativ; [13]).

Ob Ergebnisse aus Big-Data-Analysen eine Chance oder ein Risiko darstellen, entscheidet der Umgang mit den gewonnenen Erkenntnissen in Wissenschaft, Politik, Unternehmen und deren mediale Kommunikation [13, 35]. Die Akzeptanz von Big Data durch Bürger*innen hängt von der Risikokommunikation, der Datensicherheit, einer mündigen Teilhabe bzw. Entscheidungsfähigkeit der Betroffenen und dem Vertrauen in Entscheidungsträger*innen ab [5, 13]. Zwischen den Expert*innen besteht Konsens, dass digitale Bildung und Gesundheitskompetenz in der Bevölkerung Schlüsselfaktoren sein werden. Auf der Seite der Praxisakteure im Gesundheitsbereich geht es um die Data Literacy, d. h. den kompetenten Umgang mit Daten, welche bislang auch in der gesundheitswissenschaftlichen und medizinischen Ausbildung eine Herausforderung darstellt [16, 25].

In der Delphi-Studie konnten Bedingungen der Datennutzung konsentiert werden, welche u. a. bereits in der Bitkom-Leitlinie zum Einsatz von Big Data festgehalten sind [1]. Eine Bedingung ist die Anonymisierung von Daten. Diese verhindert zwar Rückschlüsse auf eine Person, jedoch nicht mögliche Stigmatisierung oder Diskriminierung bestimmter Personengruppen [15]. Auch diskriminierendes Verhalten von autonomen Kommunikationssystemen im Netz, sog. Social Bots bzw. Chatbots, ist in Experimenten mit Social-Media-Plattformen wie Twitter beobachtet worden [19]. Die Gefahr der Stigmatisierung und Diskriminierung durch Big Data wird von den Expert*innen v. a. für vulnerable und marginalisierte Gruppen gesehen. Ein wichtiger Faktor in diesem Kontext ist die ungleiche Verteilung digitaler Daten über die Bevölkerung. Ein höheres Alter, weibliches Geschlecht sowie ein niedriger Bildungsstatus sind mit einer geringeren Internetnutzung verbunden [11]. Deshalb sind aussagekräftige Daten bestimmter Gruppen, wie Sexarbeiter*innen, Kinder in prekären Familienverhältnissen, Hochbetagte, Wohnungslose oder Menschen ohne Papiere und deren Lebenswelt kaum zu gewinnen [36]. Bei Aussagen über die Repräsentativität von Big-Data-Analysen sind Verzerrungen, bspw. Populations‑/Sampling Bias oder algorithmischer Bias, zu reflektieren und ein kritischer Umgang mit den Ergebnissen erforderlich [8, 24, 25].

Nach den Erkenntnissen der Delphi-Studie bleibt zu diskutieren, ob auch bei Big-Data-basierter Forschung Partizipation, d. h. die Beteiligung der Betroffenen, möglich ist und welche Herausforderungen sich damit verbinden. Außerdem werden mögliche Kombinationen mit anderen Datenarten diskutiert. Bosse et al. [3] sehen ein Potenzial im Methodenmix mit etablierten sozialwissenschaftlichen Methoden. Im Bereich Foresight können Big-Data-basierte Systeme beim Zusammenführen heterogener Datenquellen und dem Erkennen von statistisch signifikanten Mustern unterstützen.

Die Ergebnisse der Delphi-Befragung offenbaren weiteren Forschungsbedarf zur Güte von Big-Data-Analysen, insbesondere für vulnerable und marginalisierte Gruppen, zu möglichen Verzerrungen und zum Einfluss, der im Kontext der COVID-19-Pandemie eingesetzten Big-Data-Analysen, auf die Gesundheitskompetenz und Akzeptanz der Bevölkerung.

## Limitationen

Die herausfordernde Identifikation und Gewinnung von Expert*innen deutet darauf hin, dass es in diesem Bereich noch wenig Spezialist*innen gibt. Ein ausgeglicheneres Panel, bspw. in Bezug auf das Geschlecht der Teilnehmer*innen, wäre insbesondere im Kontext der diversitätsorientierten Fragestellung wünschenswert gewesen. Das gewählte Vorgehen in dieser Studie bietet jedoch keine Möglichkeit die Hintergründe des Dissens genauer zu beleuchten.

Die Delphi-Befragung fand während der COVID-19-Pandemie statt. Dies kann die Urteile der Expert*innen beeinflusst haben. Es ist auch anzunehmen, dass eine derartige Delphi-Befragung nach der Pandemie zu veränderten Urteilen führt, gerade weil Big Data in dieser Zeit eine wichtige Rolle spielt.

## Fazit für die Praxis

- Die Expert*innen erachten die Schaffung eines wissenschaftlichen Standards und die Förderung der (digitalen) Gesundheitskompetenz in der Bevölkerung für notwendig. Ebenso muss geklärt werden, welche Akteur*innen, welche Daten, zu welchen Zwecken zukünftig analysieren dürfen.
- In den nächsten Jahren wird Big Data nach Ansicht der Expert*innen v. a. die Diagnostik und die Entwicklung individuell zugeschnittener Interventionen bzw. Therapien beeinflussen. Treibender Faktor für weitere Einsatzmöglichkeiten kann die COVID-19-Pandemie („coronavirus disease 2019“) sein, wenn bspw. durch die Corona-Warn-App datenschutzrechtliche Aspekte geklärt werden, die Bevölkerung für Big-Data-Analysen sensibilisiert ist oder die Erfassung gesundheitsbezogener Daten Teil des Alltags wird.
- Es gibt wenige Expert*innen im Bereich Big Data und Gesundheitsförderung in Deutschland. Die Integration von neuen Methoden der Datenanalyse in sozial- und gesundheitswissenschaftliche Studiengänge wäre daher wünschenswert.
